# Usefulness of modified Medium RD as a chemically defined medium for in vitro maturation of bovine oocytes

**DOI:** 10.1002/rmb2.12337

**Published:** 2020-07-06

**Authors:** Kenji Momozawa

**Affiliations:** ^1^ School of Veterinary Medicine Kitasato University Towada Aomori Japan

**Keywords:** bovine oocytes, defined medium, in vitro culture, in vitro maturation, medium RD

## Abstract

**Purpose:**

In the present study, I evaluated the usefulness of Medium RD, with mixed RPMI1640 and Dulbecco's modified Eagle's medium (1:1, v/v), as a chemically defined medium for in vitro maturation (IVM) of bovine oocytes.

**Methods:**

In vitro maturation was performed in 10 mmol/L HEPES‐buffered TCM199 (mTCM199), 10 mmol/L HEPES‐buffered Medium RD (mRD), and mTCM199 supplemented with fetal bovine serum fraction (mTCM199 + FBS fraction) that served as control. Cumulus‐oocyte complexes were matured for 24 hours in three different media supplemented with follicle‐stimulating hormone, estradiol‐17β, and polyvinylpyrrolidone. Nuclear maturation of oocytes, their developmental competence into blastocysts after in vitro fertilization (IVF) and mitochondrial distribution in oocytes were investigated.

**Results:**

There was no difference in the ratio of matured oocytes regardless of IVM media. The percentage of morula stage was higher in mRD than in mTCM199 group (*P* < .05) at 120‐144 hours after IVF, although the blastocyst rates between groups were not significantly different at 168‐216 hours. IVM in mRD increased the percentage of oocytes with diffused mitochondrial distribution compared with the immature and mTCM199 and had similar percentage of oocytes in mTCM199 + FBS fraction.

**Conclusions:**

Medium RD would be useful as a chemically defined medium for IVM of bovine oocytes.

## 1 INTRODUCTION

During in vitro maturation (IVM), oocytes undergo a series of cytoplasmic changes before the resumption of nuclear maturation, leading to variable competence of the resulting embryos.^1^ Analysis of the developmental capacity of embryos derived from in vitro matured/in vitro fertilized oocytes is a definitive way to assess the normality of maturation.^2^ In IVM of bovine oocytes, fetal bovine serum (FBS) is recommended as an oocyte maturation medium supplement.^3^ However, serum contains many unknown factors, and the quality of serum can vary greatly depending on the source (animal and/or supplier).^4^ In a previous study,^5^ we showed that the third fraction of ultracentrifuged FBS enhanced bovine oocyte development to blastocyst stage although the effective substances were not identified. Therefore, to understand the requirements for the development of immature oocytes through IVM, all products with undefined components should be eliminated from culture conditions.^6^ Moreover, Yoshioka et al^7^ indicated that a chemically defined medium is not only useful for analyzing the physical actions of substances, such as inorganic compounds, energy substrates, hormones, cytokines, and vitamins, in the development of preimplantation embryos but it is also useful for improving the reliability of media formulations, thereby increasing the reproducibility of results. However, an IVM system of bovine oocytes using a chemically defined medium has not been fully established.

In general, TCM199 is used as a basic maturation medium for bovine IVM. However, the developmental competence of bovine oocytes matured in protein‐free TCM199 was lower than that of oocytes in protein supplementation medium.^8^ Furthermore, we previously reported that the omission of protein during the maturation of bovine oocytes caused a reduction in the rate of development to the blastocyst stage.^5^ These results indicated that TCM199, as a chemically defined medium, was not sufficient for bovine IVM. In contrast, protein‐free Medium RD that contains a 1:1 (v/v) mixture of RPMI1640 and Dulbecco's modified Eagle's medium (DMEM) was shown to be superior to TCM199 for the culture of rabbit embryos.^9^ Additionally, we developed a chemically defined medium for in vitro culture (IVC) of bovine embryos using RD as a supplement for mKSOM/aa (20% RD‐mKSOM/aa, RD and mKSOM/aa, 1:4 v/v)^10^ and improved the blastocyst development by using 20% RD‐mKSOM/aa. Therefore, we assumed that Medium RD might be superior to TCM199 as an IVM medium for bovine oocytes.

A previous report showed that the redistribution of mitochondria may play multiple roles in oocyte maturation in several species.^11^ Bavister and Squirrell^12^ reported that active mitochondrial relocate during oocyte maturation or fertilization in several species. Furthermore, mitochondrial distribution in oocytes after IVM was correlated with embryo developmental competence.^13^, ^14^, ^15^, ^16^ Especially, the culture conditions of IVM affect the pattern of mitochondrial distribution in oocytes, and it correlated with the developmental competence of embryos.^13^, ^16^ Therefore, culture conditions used for IVM probably affect not only embryonic development but also mitochondrial distribution in oocytes.

In the present study, we evaluated the usefulness of Medium RD as a chemically defined medium for IVM of bovine oocytes and used TCM199 supplemented with 1.5% of the third fraction of ultracentrifuged FBS, which showed highest blastocyst development in our previous study,^5^ as control. In the first experiment, we assessed cumulus expansion and nuclear maturation. In the second experiment, we examined the effects of different maturation media on the developmental ability to the blastocyst stage after IVM. In the third experiment, to confirm the effects of maturation media on cytoplasmic maturation, we assessed the mitochondrial distribution of bovine IVM oocytes.

## 2 MATERIALS AND METHODS

### 2.1 In vitro maturation of oocytes

Bovine ovaries obtained at a local abattoir were wrapped in paper towels that were soaked in 0.9% saline and transported to the laboratory while being kept at a temperature of approximately 30°C. After confirming the absence of bovine spongiform encephalopathy, cumulus‐oocyte complexes (COCs) were aspirated from 2‐ to 6‐mm ovarian follicles into a 5‐mL disposable syringe with a 21‐gauge needle and placed in plastic Petri dishes. Fully grown oocytes with an unexpanded cumulus (more than five layers) and nonpolar ooplasm filling the perivitelline space were washed several times with maturation medium. COCs with a heterogeneous (unevenly granulated to various degrees) ooplasm were selected under a stereomicroscope at 80‐100× magnification.^17^ Selected COCs were cultured for 24 hours in 50‐μL droplets (approximately 10 COCs) of maturation medium under mineral oil (Sigma‐Aldrich Corporation) in culture dishes (35 × 10 mm, Nunc) in a CO_2_ incubator maintained at 5% CO_2_, 95% humidified air, and 39°C. IVM was performed in three different maturation media: 10 mmol/L HEPES‐buffered TCM‐199 (Nissui Pharmaceutical) supplemented with 0.3 mmol/L sodium pyruvate and 65 μg/mL dibekacin sulfate (Meiji‐Seika; mTCM199) and 10 mmol/L HEPES‐buffered Medium RD supplement with 65 μg/mL dibekacin sulfate (mRD) were used as chemically defined media. Medium RD is a 1:1 (v/v) mixture of RPMI1640 (Sigma‐Aldrich) and DMEM (low glucose; Sigma‐Aldrich).^9^ The mTCM199 supplemented with 1.5% of the third fraction of ultracentrifuged FBS (Hyclone; Thermo Fisher Scientific Inc; mTCM199 + FBS fraction) was used as control. The third fraction of FBS was prepared by ultracentrifugation of FBS at 220 000 *g* for 48 hours at 4°C as reported.^5^ All maturation media were further supplemented with 0.12 IU/mL follicle‐stimulating hormone (FSH, Antrin R‐10; Kawasaki‐Mitaka Seiyaku), 1 μg/mL estradiol‐17β (Sigma‐Aldrich), and 3 mg/mL polyvinylpyrrolidone (PVP, MW 40 000; Sigma‐Aldrich).

### 2.2 Evaluation of cumulus expansion

The evaluation of cumulus expansion was conducted according to Funahashi and Day.^18^ Briefly, a single COC was introduced into a 50‐μL droplets. Then, cumulus cell expansion was evaluated by the measurement of cumulus diameters after 0 and 24 hours of IVM. The diameter of a single COC was determined by measuring the minimum and maximum diameters with an eyepiece micrometer under an inverted microscope at 100× magnification and by calculating the mean diameter as (minimum diameter + maximum diameter) × 2^−1^.

### 2.3 Evaluation of nuclear maturation

At the end of IVM, COCs were removed from the droplet and treated with 0.1% hyaluronidase dissolved in Dulbecco's phosphate‐buffered saline (PBS) supplemented with 1.0 mg/mL PVP and then denuded of cumulus cells by gentle pipetting. Subsequently, oocytes were mounted on glass slides, fixed with 25% (v/v) acetic alcohol for 48 hours, stained with 1% (w/v) orcein in 45% (v/v) acetic acid, and examined under a Nomarski interference‐contrast microscope at 400× magnification. Oocytes with a metaphase plate and one polar body (PB) were classified as metaphase II (M‐II).

### 2.4 In vitro fertilization

In vitro fertilization (IVF) was performed according to Momozawa and Fukuda.^10^ Briefly, one straw (0.5 mL/straw) of frozen semen of Japanese Black cattle was thawed in a water bath at 39°C for 20 seconds, suspended in 5 mL of fertilization medium with 10 mmol/L caffeine (sodium benzoate, Sigma‐Aldrich), and washed twice by centrifugation at 700 *g* for 5 min. The fertilization medium was modified BO (HEPES‐BO, bovine serum albumin‐free).^17^ The washed spermatozoa were resuspended in 0.5 mL of fertilization medium supplemented with 10 mmol/L caffeine. The sperm suspension was diluted 1:1 with fertilization medium supplemented with 1.0 mg/mL penicillamine (Sigma‐Aldrich), 0.5 mmol/L methyl‐β‐cyclodextrin (Sigma‐Aldrich), 1.0 mmol/L citrate (sodium citrate‐trisodium salt, Kanto Chemical Co., Inc), and 2 mg/mL PVP. The spermatozoa were preincubated for 4 hours in a CO_2_ incubator maintained at 5% CO_2_, 95% humidified air, and 39°C. The sperm concentration at preincubation was ~2 × 10^7^ sperm/mL. After the 4‐hour preincubation, 5 μL of sperm suspension was introduced into each 40‐μL droplet of fertilization medium with 1.0 mg/mL PVP. Eventually, the sperm concentration at insemination was about 1000 cells/μL. At 30 min after the introduction of sperm, COCs after IVM were introduced into the droplets of sperm suspension (about 10 oocytes/drop). In all experiments, spermatozoa and oocytes were coincubated for 6 hours in a CO_2_ incubator maintained at 5% CO_2_, 95% humidified air, and 39°C.

### 2.5 In vitro embryo culture

Six hours after insemination, oocytes were treated with 10 units/mL hyaluronidase dissolved in the fertilization medium with 1.0 mg/mL PVP and then denuded of cumulus cells by gentle pipetting. Subsequently, presumptive zygotes were cultured for 216 hours in 50 μL of culture medium covered with mineral oil under a gas phase of 5% CO_2_, 5% O_2_, and 90% N_2_ with high humidity at 39°C. The embryo culture medium used as a chemically defined medium in the present study was 20% RD‐mKSOM/aa.^10^ Each droplet contained approximately 10 presumptive zygotes. Oocytes that did not progress to the 2‐cell stage at 48 hours after insemination were removed from the drops containing developing embryos. At 96 hours after insemination, embryos at the 8‐cell stage or a more advanced stage were transferred to a fresh culture medium supplemented with 10 μmol/L β‐mercaptoethanol (β‐ME; Sigma‐Aldrich). Between 120 and 144 hours after insemination, embryos at the morula stage or a more advanced stage were transferred to a fresh culture medium supplemented with 50 μmol/L β‐ME. The morphological quality of embryos at blastocyst stage was evaluated following the criteria of IETS manual.^19^ Namely, blastocysts of quality Code 1 with stage Codes 6 and 7 were treated as embryos developed to blastocyst stage.

### 2.6 Evaluation of the mitochondrial distribution in oocytes

**FIGURE 1:**
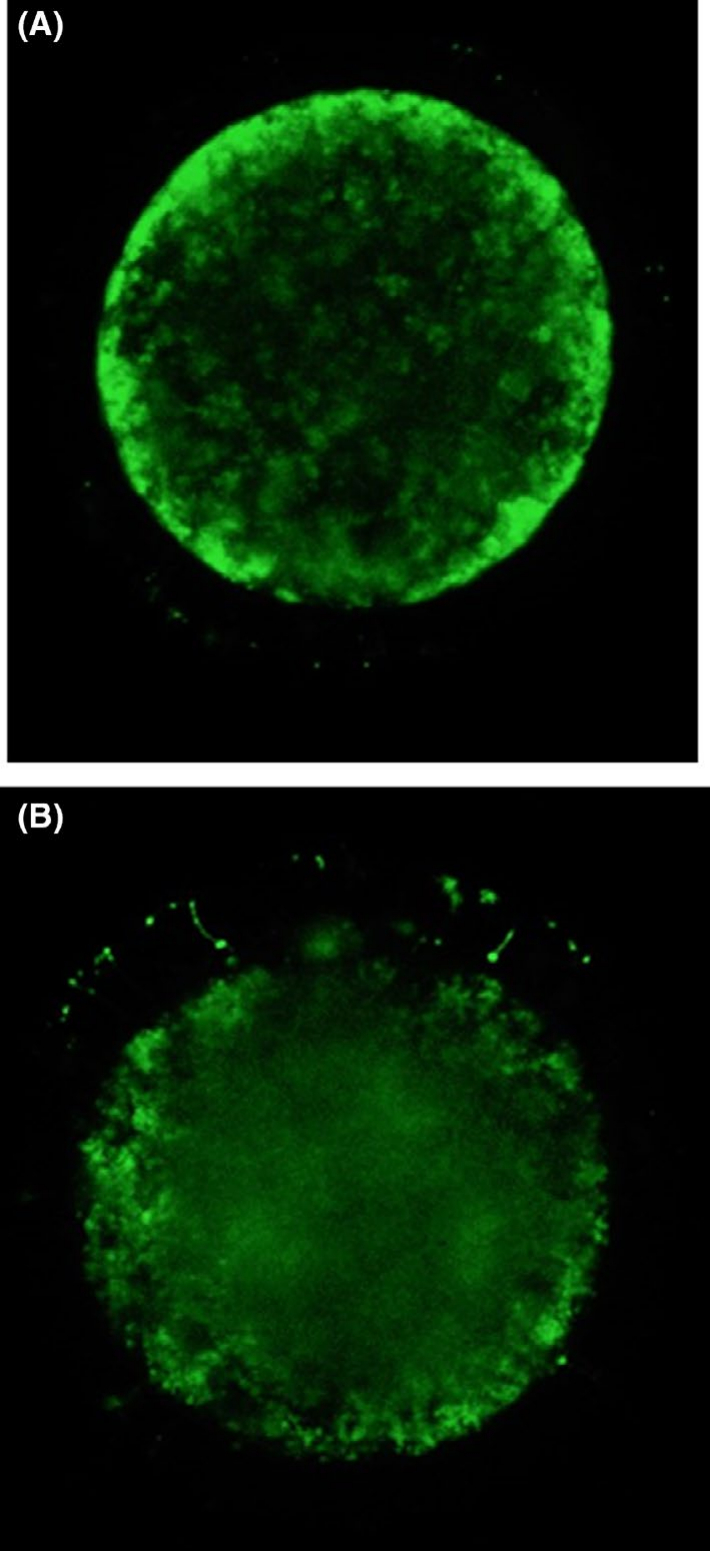
Distribution of mitochondria in bovine oocytes. Laser scanning images were taken at the equatorial plane of oocytes at 400× magnification. Mitochondria stained by rhodamine 123 appear in green color. A, Mitochondria located in the peripheral cytoplasm rather than in the central cytoplasm. B, Mitochondria diffused to the inner region of ooplasm with evenly or unevenly

Denuded oocytes by gentle pipetting were incubated with 10 μg/mL rhodamine 123 (Sigma‐Aldrich) in PBS for 15 min, mounted on glass slides, and immediately observed using an LSM‐710 confocal laser scanning microscope (Zeiss). The fluorescence was observed using a 514‐nm excitation line and a 524‐ to 560‐nm emission filter. Mitochondrial distribution in oocytes was classified according to Brevini et al^16^ with some modifications. Peripheral mitochondrial distribution was characterized by the location of mitochondria beneath the plasma membrane (Figure 1A). Diffused distribution was characterized by the homogeneous or heterogeneous (containing semi‐peripheral) location of mitochondria throughout the cytoplasm (Figure 1B).

### 2.7 Experimental design

#### 2.7.1 Experiment 1

After IVM culture, 168 COCs were submitted to the measurement of their diameter (n = 70) and evaluated the nuclear status (n = 98).

#### 2.7.2 Experiment 2

After IVM culture of 455 COCs, IVF and in vitro embryo culture (IVC) were carried out. Cleavage and development to the 8‐cell or a more advanced stage, the morula stage, and the blastocyst stage at days 7, 8, and 9 were evaluated at 48, 96, 120‐144, 168, 192, and 216 hours after insemination, respectively.

#### 2.7.3 Experiment 3

After IVM, 77 oocytes at the M‐II stage were selected under a stereomicroscope according to the presence of the first PB and stained by rhodamine 123. Then, mitochondrial distribution in oocytes was evaluated. Immature oocytes (n = 33) immediately after collection from ovaries were used as control.

### 2.8 Statistical analysis

Difference in cumulus expansion was analyzed by a one‐way ANOVA followed by Tukey's multiple comparison test. Differences in nuclear maturation rates and distribution of mitochondria between groups were analyzed using the chi‐square test. Data for embryonic development were subjected to an arcsine transformation. The effect of treatment on embryonic development until morula stage was analyzed by a one‐way ANOVA followed by Tukey's multiple comparison test. The effects of treatment and culture period (days 7, 8, and 9) on the blastocyst development were analyzed by a two‐way ANOVA followed by Tukey's multiple comparison test.

## 3 RESULTS

### 3.1 Experiment 1: Effects of different IVM media on cumulus expansion and nuclear maturation

As shown in Table 1, all groups showed good cumulus expansion after IVM. There was no difference in the mean diameters of COCs after 24 hours of culture between the groups. The results of nuclear maturation are presented in Table 2. The ratios of matured (M‐II stage) oocytes after IVM were not different among the groups.

**TABLE 1:** Effects of different maturation media on cumulus expansion

In vitro maturation medium^a^	No. of oocytes examined	Cumulus‐oocyte complexe diameters (µm) at each culture duration (h)	
		0	24
mTCM199	17	397.1 ± 21.2	659.7 ± 35.9
mRD	36	424.0 ± 19.6	727.8 ± 35.4
mTCM199 + FBS fraction	17	378.8 ± 14.4	709.7 ± 29.3

Note

Values are means ± standard deviations.

^a^ Before use, all of the listed basic media were further supplemented with 0.12 IU/mL follicle‐stimulating hormone, 1 μg/mL estradiol‐17β, and 3 mg/mL polyvinylpyrrolidone.

**TABLE 2:** Effect of different maturation media on nuclear maturation after in vitro maturation (IVM)

IVM medium^a^	No. of oocytes examined (replicates)	No. of oocytes reached			
		M‐I	A‐I	T‐I	M‐II (%)
mTCM199	34 (3)	2	0	0	32 (94.1)
mRD	32 (2)	0	0	0	32 (100)
mTCM199 + FBS fraction	32 (3)	4	0	0	28 (87.5)

Abbreviations: A‐I, anaphase I; M‐I, metaphase I; M‐II, metaphase II; T‐I, telophase I.

^a^ See Table 1.

### 3.2 Experiment 2: Effects of different IVM media on in vitro developmental ability to the blastocyst stage after IVF

As shown in Table 3, there was no significant difference in the rates of ≥2‐ and ≥8‐cell stages between the groups; however, the percentage of morula stage was higher in the mRD than in the mTCM199 group (*P* < .05). Analysis by a two‐way ANOVA showed the effects of IVM media and culture period on the blastocyst development (*P* = .07 and .06, respectively). Namely, the blastocyst rate up to day 9 tended to be higher in mTCM199 + FBS fraction group than in mTCM199 group (*P* = .07); however, those were similar between mRD and mTCM199 + FBS fraction groups (*P* = .64). In addition, the blastocyst rate in mRD group was significantly higher at days 8 and 9 than at day 7 (*P* < .05).

**TABLE 3:** Effects of different maturation media on in vitro development of bovine in vitro maturation (IVM)‐in vitro fertilization oocytes

IVM medium^†^	No. of oocytes examined (replicates)	Numbers (%) of oocytes developed to					
		≥2 cells	≥8 cells	Morula	Blastocyst at		
					Day 7	Day 8	Day 9
mTCM199	133 (7)	108 (80.3 ± 8.4)	88 (64.9 ± 17.3)	66 (49.0 ± 13.5)^b^	56 (40.8 ± 13.5)	58 (42.4 ± 14.6)	59 (43.3 ± 15.3)
mRD	165 (6)	146 (87.9 ± 7.4)	124 (74.2 ± 12.4)	106 (63.5 ± 4.6)^a^	65 (38.1 ± 9.2)^x^	81 (49.5 ± 5.7)^y^	89 (53.5 ± 5.7)^y^
mTCM199 + FBS fraction	157 (7)	139 (87.7 ± 6.9)	119 (76.6 ± 7.6)	95 (61.4 ± 9.0)^ab^	69 (46.0 ± 10.1)	77 (51.2 ± 9.9)	81 (53.4 ± 9.5)

Note

Values are means ± standard deviations.

^a,b^ Values in the same column with different superscripts are significantly different (*P* < .05).

^x,y^ Values in the same raw with different superscripts are significantly different (*P* < .05).

Development to 2‐cell, 8‐cell or a more advanced stage, morula stage, and blastocyst stages at days 7, 8, and 9 were evaluated at 48, 96, 120‐144, 168, 192, and 216 h after insemination, respectively.

^†^ See Table 1.

### 3.3 Experiment 3: Effects of different IVM media on mitochondrial distribution in matured oocytes

The results for mitochondrial distribution are presented in Table 4. Most of the oocytes before IVM (72.7%) had a peripheral mitochondrial distribution, whereas the remaining 27.3% showed a diffused mitochondrial distribution. IVM in mRD and mTCM199 + FBS fraction significantly increased the percentage of the oocytes with a diffused mitochondrial distribution compared with the immature and matured mTCM199 groups (*P* < .01).

**TABLE 4:** Mitochondrial distribution in bovine oocytes before and after in vitro maturation (IVM)

Culture duration (h)	IVM medium^†^	No. of oocytes examined (replicates)	Mitochondrial distribution (%)	
			Peripheral	Diffused
0	—	33 (3)	24 (72.7)	9 (27.3)^a^
24	mTCM199	25 (2)	16 (64.0)	9 (36.3)^a^
	mRD	25 (3)	4 (16.0)	21 (84.0)^b^
	mTCM199 + FBS fraction	22 (3)	4 (18.2)	18 (81.8)^b^

Note

^a,b^ Values in the same column with different superscripts are significantly different (*P* < .01).

^†^ See Table 1.

## 4 DISCUSSION

A chemically defined medium would be useful for analyzing promoters or inhibitors during the IVM of bovine oocytes. An advanced chemically defined medium for IVM of pig oocytes, named POM, has been developed.^7^ Although IVM of bovine oocytes is a widely used technique for in vitro production (IVP) of embryos, an advanced chemically defined medium for IVM has not been fully established. In general, TCM199 has been used as a basic maturation medium for bovine IVM. However, the developmental competence of bovine oocytes matured in protein‐free TCM199 was lower than that of oocytes in a protein supplementation medium.^5^, ^8^ In contrast, protein‐free Medium RD (RPMI1640 and DMEM, 1:1 v/v) was shown to be superior to TCM199 for the culture of rabbit embryos.^9^ Therefore, we hypothesized that Medium RD could be superior to TCM199 as an IVM medium for bovine oocytes.

Furnus et al^20^ reported that optimal cumulus expansion in vitro plays an important role in subsequent embryonic development up to the blastocyst stage. Therefore, we examined the effects of IVM media on cumulus expansion in the present study. We found no differences in the degree of cumulus expansion and the percentage of oocytes reaching the M‐II stage among the groups. Our results were consistent with the results of a previous study showing that oocyte maturation media or their supplements did not affect the rate of nuclear maturation.^21^ Previous reports indicated that supplementation of FSH to the maturation medium promoted the cumulus expansion of bovine oocytes.^6^, ^22^ In the present study, the enhancement of cumulus expansion was probably due to the supplementation of FSH for all groups regardless of the difference of the maturation media.

As shown in Table 3, the rate of development to the morula stage in mRD group was higher than protein‐free TCM199 and similar to TCM‐199 supplemented with FBS fraction, and the blastocyst rate in mRD group was similar to that in mTCM199 + FBS fraction group, which showed the higher development to blastocyst stage compared with mTCM199 group. In the present study, the percentage of blastocyst in mRD group increased from day 7 to days 8 and 9, although the blastocyst rates in mTCM199 and mTCM199 + FBS fraction groups were stable between days 7 to 9. One of the reason of increasing blastocyst rate in mRD group may be the evaluation method of blastocysts. In the present study, I evaluated the blastocysts based on the morphological criteria Code 1 in IETS manual^22^ and judged as blastocyst if the embryo had clear blastocoel. At day 7, blastocysts derived from mRD group may have a large number of cells in inner cell mass (ICM) and early blastocysts might be judged as morulae due to small blastocoel compared with blastocysts derived from other groups, because the expanded blastocysts in mRD group showed higher rate of ICM/total cells in blastocysts (Appendix S1 and Table S1). Therefore, these results indicate that mRD improves the developmental competence of bovine oocytes at least the same as FBS addition to IVM medium and may be useful for a chemically defined IVM medium. Unexpectedly, the oocytes matured with mTCM199 without protein supplementation showed a relatively high developmental competence in this study. This result is contradictory to the results of our previous study showing that the developmental competence of bovine oocytes matured in protein‐free TCM199 was lower than that of oocytes matured in a protein supplementation medium.^5^ These differing results are likely related to different media used for IVC. Previously, we developed a chemically defined medium for IVC of bovine embryos named 20% RD‐mKSOM/aa.^10^ In the present study, 20% RD‐mKSOM/aa was used as an IVC medium, whereas modified TCM199 containing bovine serum albumin was used as an IVC medium in the previous study.^5^ The reason for the relatively high developmental competence with mTCM199 might be due to the use of an advanced IVC medium in the present study.

Krisher et al^11^ reported that the redistribution of mitochondria may play multiple roles in oocyte maturation in several species. In cattle,^14^, ^23^, ^24^ pigs,^16^, ^25^ and humans,^15^ the pattern of mitochondrial distribution changes during oocyte maturation, and the mitochondria localize more toward the center of the matured oocyte. The changes in mitochondrial distribution in these reports were consistent with the results of this study. Additionally, in the present study, the rate of mitochondria distributed throughout the cytoplasm of oocytes that matured with mRD was significantly higher than that of oocytes matured with mTCM199 without protein supplementation. Bavister and Squirrell^12^ suggested that the distribution pattern and location of mitochondria in bovine oocytes appear to correlate with developmental competence. Moreover, the culture conditions of IVM affect the pattern of mitochondrial distribution in oocytes, and this pattern is correlated with the developmental competence of embryos.^13^, ^16^ Therefore, the results of the present study indicated that mitochondrial distribution through the ooplasm in bovine oocytes after IVM was linked to high developmental competence to the blastocyst stage.

Although the effective substance(s) contained in mRD were not clarified in the present study, the key substance in mRD is probably inositol. Carney and Foote^9^ reported that a characteristic of Medium RD is the concentration of inositol, which is more than 400‐times higher in Medium RD (117 µmol/L) than in TCM199 (0.28 μmol/L). Moreover, it was reported that inositol was beneficial for embryonic development in rabbits,^26^, ^27^ hamsters,^28^ and cattle.^29^, ^30^ However, to the best of our knowledge, there have been few studies on the effect of inositol on IVM and subsequent embryonic development in cattle. Chiu et al^31^ suggested that follicles containing good‐quality oocytes had higher concentrations of myo‐inositol in follicular fluid, probably due to the intricate relationship between myo‐inositol and inositol phosphates in the phosphatidylinositol cycle activation for oocyte maturation. Additionally, they reported that the exposure of mouse germinal vesicle oocytes to myo‐inositol (30 mmol/L) during IVM enhanced meiotic maturation and the subsequent developmental potential of oocytes following fertilization.^32^ Coticchio and Fleming^33^ suggested that inositol triphosphate generated in cumulus cells may transfer to the oocyte via intercellular communication in mouse cumulus‐enclosed oocytes. On the other hand, Hynes et al^30^ showed that incorporation of inositol into inositol phosphates was not detected until blastocyst formation at day 7 although uptake of inositol occurred at 2‐cell embryo stage in cattle. These results suggest that uptake of inositol into cumulus cells during IVM in mRD group promotes the development of bovine embryos from one/two‐cell to the morula stage in the present study. In addition, Chiu et al^32^ showed that myo‐inositol addition to mouse IVM medium caused spontaneous calcium release from endoplasmic reticulum. It may be one of the causes of the differences in the mitochondrial distribution between the experimental groups in the present study, because the calcium release and mitochondrial activity in mammalian oocytes are closely related.^34^ In a further study, the role of inositol in IVM of bovine oocytes should be investigated in detail.

In conclusion, the present study suggested that Medium RD would be useful as a chemically defined medium for bovine oocyte maturation in vitro. This defined IVM medium can be used to provide more precise information to better understand the requirements for IVM of bovine oocytes. Additionally, the present study established a successful IVP system of bovine embryos using chemically defined media for IVM, IVF, and IVC. In a future study, we will evaluate the full‐term development of embryos produced with this novel IVP system.

## DISCLOSURES

*Conflict of interest*: The author has no conflicts of interest to declare. *Human/Animal rights:* This article does not contain any studies with human and animal subjects performed by the author.

## Supporting information

App S1

Table S1

## References

[1] Moor RM , Mattioli M , Ding J , Nagai T . Maturation of pig oocytes *in vivo* and *in vitro* . J Reprod Fertil Suppl. 1990;40:197‐210. 2192038

[2] Rose TA , Bavister BD . Effects of oocyte maturation medium on *in vitro* development of *in vitro* fertilized bovine embryos. Mol Reprod Dev. 1992;31:72‐77. 1562330 10.1002/mrd.1080310113

[3] Parrish JJ . Application of *in vitro* fertilization to domestic animal In: Wasserman PM, ed. Elements of Mammalian fertilization, Vol. II Boca Raton, FL: CRC Press; 1991:111‐128.

[4] Pinyopummintr T , Bavister BD . Development of bovine embryos in a cell‐free culture medium: Effects of type of serum, timing of its inclusion and heat inactivation. Theriogenology. 1994;41:1241‐1249. 16727477 10.1016/0093-691x(94)90481-w

[5] Momozawa K , Fukuda Y . Effects of fractions of bovine follicular fluid and fetal bovine serum as supplements to maturation medium on *in vitro* development of *in vitro* fertilized bovine embryo. J Mamm Ova Res. 2011;28:68‐74.

[6] Ali A , Sirard MA . Effect of the absence or presence of various protein supplements on further development of bovine oocytes during *in vitro* maturation. Biol Reprod. 2002;66:901‐905. 11906907 10.1095/biolreprod66.4.901

[7] Yoshioka K , Suzuki C , Onishi A . Defined system for *in vitro* production of porcine embryos using a single basic medium. J Reprod Dev. 2008;55:208‐213. 10.1262/jrd.20001 18408352

[8] Raty M , Ketoja E , Pitkanen T , Ahola V , Kananen K , Peippo J *In vitro* maturation supplements affect developmental competence of bovine cumulus‐oocyte complexes and embryo quality after vitrification. Cryobiology. 2011;63:245‐255. 21985767 10.1016/j.cryobiol.2011.09.134

[9] Carney EW , Foote RH . Improved development of rabbit one‐cell embryos to the hatching blastocyst stage by culture in a defined, protein‐free culture medium. J Reprod Fert. 1991;91:113‐123. 10.1530/jrf.0.0910113 1995842

[10] Momozawa K , Fukuda Y . Establishment of an advanced chemically defined medium for early embryos derived from *in vitro* matured and fertilized bovine oocytes. J Reprod Dev. 2011;57:681‐689. 21804301 10.1262/jrd.11-039h

[11] Krisher RL , Brad AM , Herrick JR , Sparman ML , Swain JE . A comparative analysis of metabolism and viability in porcine oocytes during *in vitro* maturation. Anim Reprod Sci. 2007;98:72‐96. 17110061 10.1016/j.anireprosci.2006.10.006

[12] Bavister BD , Squirrell JM . Mitochondrial distribution and function in oocytes and early embryos. Hum Reprod. 2000;15(Suppl 2):189‐198. 10.1093/humrep/15.suppl_2.189 11041524

[13] Krisher RL , Bavister BD . Responses of oocytes and embryos to the culture environment. Theriogenology. 1998;49:103‐114. 10732124 10.1016/s0093-691x(97)00405-6

[14] Stojkovic M , Machado SA , Stojkovic P , et al. Mitochondrial distribution and adenosine triphosphate content of bovine oocytes before and after *in vitro* maturation: correlation with morphological criteria and developmental capacity after *in vitro* fertilization and culture. Biol Reprod. 2001;64:904‐909. 11207207 10.1095/biolreprod64.3.904

[15] Wilding M , Dale B , Marino M , et al. Mitochondrial aggregation patterns and activity in human oocytes and preimplantation embryos. Human Reporod. 2001;16:909‐917. 10.1093/humrep/16.5.909 11331637

[16] Brevini TAL , Vassena R , Francisci C , Gandolfi F . Role of adenosine triphosphate, active mitochondria, and microtubules in the acquisition of developmental competence of parthenogenetically activated pig oocytes. Biol Reprod. 2005;72:1218‐1223. 15659704 10.1095/biolreprod.104.038141

[17] Momozawa K , Fukuda Y *In vitro* maturation and *in vitro* fertilization of bovine with heterogeneous ooplasm. Anim Sci Technol. 1995;66:605‐609. (In Japanese).

[18] Funahashi H , Day BN . Effects of the duration of exposure to hormone supplements on cytoplasmic maturation of pig oocytes *in vitro* . J Reprod Fert. 1993;98:179‐185. 10.1530/jrf.0.0980179 8345463

[19] Stringfellow DA , Givens MD . Manual of International Embryo Transfer Society (IETS). Savoy, IL: IETS; 2009.

[20] Furnus CC , de Matos DG , Moses DF . Cumulus expansion during *in vitro* maturation of bovine oocytes: relationship with intracellular glutathione level and its role on subsequent embryo development. Mol Reprod Dev. 1998;51:76‐83. 9712320 10.1002/(SICI)1098-2795(199809)51:1<76::AID-MRD9>3.0.CO;2-T

[21] Russell DF , Baqir S , Bordignon J , Betts DH . The impact of oocyte maturation media on early bovine embryonic development. Mol Reprod Dev. 2006;73:1255‐1270. 16865717 10.1002/mrd.20553

[22] Kobayashi K , Yamashita S , Hoshi H . Influence of epidermal growth factor and transforming growth factor‐α on *in vitro* maturation of cumulus cell‐enclosed bovine oocytes in a defined medium. J Reprod Fertil. 1994;100:439‐446. 8021861 10.1530/jrf.0.1000439

[23] Tarazona AM , Rodriguez JI , Restepo LF , Olivera‐Angel M . Mitochondrial activity, distribution and segregation in bovine oocytes and embryos produced *in vitro* . Reprod Domest Anim. 2006;41:5‐11. 16420320 10.1111/j.1439-0531.2006.00615.x

[24] Somfai T , Inaba Y , Watanabe S , Geshi M , Nagai T . Follicular fluid supplementation during *in vitro* maturation promotes sperm penetration in bovine oocytes by enhancing cumulus expansion and increasing mitochondrial activity in oocytes. Reprod Fertil Dev. 2012;24:743‐752. 22697124 10.1071/RD11251

[25] Sun QY , Wu GM , Lai L , et al. Translocation of active mitochondria during pig oocyte maturation, fertilization and early embryo development *in vitro* . Reproduction. 2001;122:155‐163. 11425340

[26] Kane MT . Effects of the putative phospholipid precursors, inositol, choline, serine and ethanolamine, on formation and expansion of rabbit blastocysts *in vitro* . J Reprod Fertil. 1989;87:275‐279. 2516133 10.1530/jrf.0.0870275

[27] Fahy MM , Kane MT . Inositol stimulates DNA and protein synthesis, and expansion by rabbit blastocysts *in vitro* . Hum Reprod. 1992;7:550‐552. 1522201 10.1093/oxfordjournals.humrep.a137688

[28] Kane MT , Bavister BD . Vitamin requirements for development of eight‐cell embryos to hatching blastocysts *in vitro* . Biol Reprod. 1988;39:1137‐1143. 3219385 10.1095/biolreprod39.5.1137

[29] Holm P , Booth PJ , Schmidt MH , Greve T , Callesen H . High bovine blastocyst development in a static *in vitro* production system using SOFaa medium supplemented with sodium citrate and myo‐inositol with or without serum‐proteins. Theriogenology. 1999;52:683‐700. 10734366 10.1016/S0093-691X(99)00162-4

[30] Hynes AC , Sreenan JM , Kane MT . Uptake and incorporation of myo‐inositol by bovine preimplantation embryos from two‐cell to early blastocyst stages. Mol Reprod Dev. 2000;55:265‐269. 10657045 10.1002/(SICI)1098-2795(200003)55:3<265::AID-MRD4>3.0.CO;2-6

[31] Chiu TTY , Rogers MS , Law ELK , Briton‐Jones CM , Cheung LP , Haines CJ . Follicular fluid and serum concentrations of myo‐inositol in patients undergoing IVF: relationship with oocyte quality. Hum Reprod. 2002;17:1591‐1596. 12042283 10.1093/humrep/17.6.1591

[32] Chiu TTY , Rogers MS , Briton‐Jones C , Haines CJ . Effects of myo‐inositol on the *in‐vitro* maturation and subsequent development of mouse oocytes. Hum Reprod. 2003;18:408‐416. 12571181 10.1093/humrep/deg113

[33] Coticchio G , Fleming S . Inhibition of phosphoinositide metabolism or chelation of intracellular calcium blocks FSH‐induced but not spontaneous meiotic resumption in mouse oocytes. Dev Biol. 1998;203:201‐209. 9806784 10.1006/dbio.1998.9021

[34] Yeste M , Jones C , Amdani SN , Patel S , Coward K . Oocyte activation deficiency: a role for an oocyte contribution? Hum Reprod Update. 2015;22:23‐47. 26346057 10.1093/humupd/dmv040

