# Supplementary material for: Usefulness of modified Medium RD as a chemically defined medium for in vitro maturation of bovine oocytes
Source: Reprod Med Biol. 2020 Jul 6;19(4):365–71. doi: 10.1002/rmb2.12337 (PMC7542018; doi:10.1002/rmb2.12337)
Supplement: Supplementary file 1 — App S1 [file RMB2-19-365-s001.docx]

**Appendix S1. Evaluation of cell numbers in expanded blastocysts derived from different IVM media**

*Methods:*

Cell numbers in blastocysts

Expanded blastocysts judged as quality Code 1 based on the criteria of IETS were used in the experiment. The quality of blastocysts at days 7 and 8 was analyzed by differential cell staining of the inner cell mass (ICM) and the trophectoderm (TE) cells according to the previous study [Thomas et al., Reprod Biomed Online, 2001]. Briefly, TE cells of blastocysts were permeabilized and stained by incubating embryos in PBS containing 0.2% Triton X-100 and 100 μg/ml propidium iodide (PI) for 60 sec at 39°C. ICM cells of blastocysts were then counterstained via incubating the embryos in 25 μg/ml bisbenzimide (Hoechst 33342) dissolved in ethanol for 2 to 3 h at 4°C. The blastocysts were washed once in glycerol before being mounted on a glass slide with the same medium. Under an epifluorescence microscope, ICM cell nuclei labeled with bisbenzimide appeared blue, and TE cell nuclei labeled with PI and bisbenzimide appeared pink.

*Results:*

As shown in Supplementary Table, there were no significant differences in total and ICM cell numbers in blastocysts between three groups. However, the ratio of ICM/total cells in blastocysts derived from mRD group was higher than that from mTCM199 group (P = 0.05, Tukey’s multiple comparison test).
