# Supplementary material for: Usefulness of modified Medium RD as a chemically defined medium for in vitro maturation of bovine oocytes
Source: Reprod Med Biol. 2020 Jul 6;19(4):365–71. doi: 10.1002/rmb2.12337 (PMC7542018; doi:10.1002/rmb2.12337)
Supplement: Supplementary file 2 — Table S1 [file RMB2-19-365-s002.docx]

Table S1. Effects of different maturation media on the cell numbers in blastocysts

| IVM medium | No. of oocytes examined |  | No. of cells/blastocysts | |  | Ratio of ICM/total cells |
| --- | --- | --- | --- | --- | --- | --- |
|  |  |  | Total | ICM ^a^ |  |  |
| mTCM199 | 9 |  | 146.3 ± 9.6 | 42.7 ± 5.2 |  | 29.0 ± 2.6 |
| mRD | 6 |  | 154.2 ± 23.0 | 62.5 ± 12.6 |  | 40.5 ± 4.5 |
| mTCM199+FBS fraction | 6 |  | 141.4 ± 10.9 | 43.8 ± 5.1 |  | 31.4 ± 3.4 |

Values are means ± standard deviations.

^a^ ICM: inner cell mass.
